# Commentary: Plasma angiotensin II is increased in critical coronavirus disease 2019

**DOI:** 10.3389/fcvm.2022.1012452

**Published:** 2022-10-17

**Authors:** Dirk van Lier, Matthijs Kox, Peter Pickkers

**Affiliations:** Department of Intensive Care Medicine and Radboud Center for Infectious Diseases (RCI), Radboud University Medical Center, Nijmegen, Netherlands

**Keywords:** angiotensin converting enzyme, renin angiotensin aldosterone system, COVID-19, enzyme-linked immunosorbent assay (ELISA), liquid chromatography–mass spectrometry

## Introduction

With great interest, we read the study by Camargo et al. on systemic angiotensin responses in critically ill COVID-19 patients (1). Ever since it became known that angiotensin converting enzyme-2 (ACE2) is the functional receptor for SARS-CoV-2, there has been extensive debate about the influence of ACE2 expression and systemic angiotensin responses on COVID-19 susceptibility and outcome. In the past year, multiple independent research groups have reported data on angiotensin metabolism in critically ill COVID-19 patients, albeit with results that sometimes appear conflicting.

The main effect of classical renin angiotensin aldosterone system (RAAS) activation is the generation of angiotensin-II (Ang-II) by angiotensin converting enzyme (ACE) (2). Contrary, non-classical RAAS activation results in cleavage of angiotensin-II by ACE2 to form angiotensin 1–7 (Ang-1–7), which directly counteracts the classical angiotensin-II/ACE pathway (2). Camargo et al. report increased plasma levels of Ang-II in critically ill COVID-19 patients, compared to patients that presented with less severe COVID-19. We want to raise two comments related to their study;

1. The enzyme-linked immunosorbent assays (ELISAs) employed by the authors suffer from known methodological issues, resulting in artificially high angiotensin metabolite levels.
2. The study's emphasis on classical RAAS alterations prevents the authors from reaching important conclusions about non-classical RAAS alterations, which appear to be more pronounced in COVID-19 patients.

## Discussion

### Methodological difficulties in assessing systemic angiotensin responses

The accurate evaluation of angiotensin responses is hampered by low levels of peptides present in the circulation combined with rapid metabolism during sample processing, because enzymes like ACE, ACE2 and neprilysin remain active (3). Therefore, differences in sample processing in the absence of enzyme inhibitor cocktails may yield artificially high peptide values that do not reflect endogenous content (4). The added methodological difficulties of obtaining samples in a critically ill population in strict isolation may further complicate timely processing, thereby increasing the risk of measuring artificially high peptide levels in these patients even more.

Because of their sensitivity to detect metabolites in the picomolar range, radioimmuno-assays have long served as the gold standard for measuring angiotensin pathway metabolites (3). In this method, RAAS-enzyme inhibition is rapidly applied during sampling, ideally by using vacutainers pre-filled with enzyme inhibitor cocktails. More recently, liquid chromatography–mass spectrometry (LC-MS)-based assays has been developed. Combined with enzyme inhibitor cocktails, these assays are equally sensitive as radio-immuno-assays, reflecting real time levels of circulating angiotensin metabolites with high levels of measurement reproducibility (5). However, as few laboratories possess the expertise and specialized equipment necessary for both of these approaches, costs remain high. This explains why ELISAs such as used by Camargo et al. are increasingly used, as both the costs and required specialized equipment of this method are comparatively minimal.

However, since Camargo et al. made no use of enzyme inhibitors, their measurements are unlikely to reflect true endogenous angiotensin metabolite contents. Furthermore, apart from falsely high levels due to ongoing metabolism after sampling, other specific concerns have recently been raised about the accuracy of ELISAs to assess angiotensin levels (4). In a methodological study, two commercially available ELISA kits were found to measure artificially high levels of Ang-II and Ang-1–7 compared to gold standard methods, even when immediate enzyme inhibition was applied (4). Subsequently, it was demonstrated that these measurement artifacts are most likely explained by assay cross-reactivity with other (unknown) peptides present in the plasma (4).

**Figure 1:**
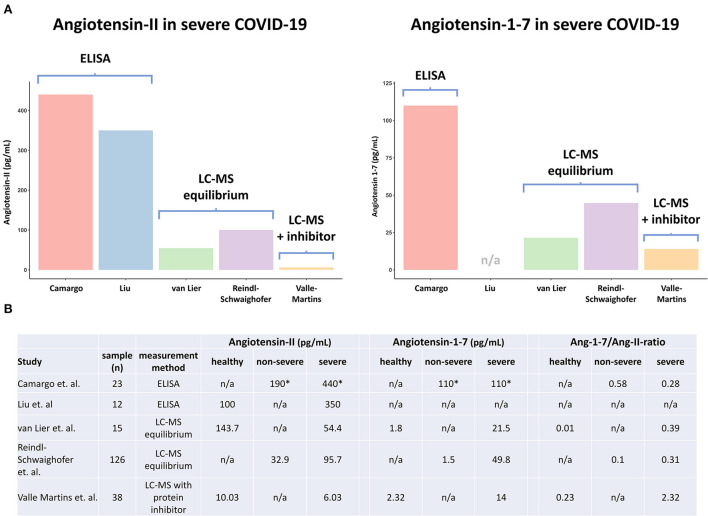
Overview of angiotensin metabolite measurements in COVID-19 patients and healthy volunteers in different studies. ELISA, enzyme-linked immunoassay; LC-MS, liquid chromatography–mass spectrometry; LC-MS equilibrium, measurement method without protein inhibitor cocktail, that assesses RAAS enzymes in the circulatory compartment; LC-MS with inhibitor cocktail, measurement method that assesses true circulating levels of metabolites by direct inhibition of enzymes during sample withdrawal; Ang-1–7/Ang-II-ratio, Angiotensin-1-7 Angiotensin-II ratio. *Exact levels of angiotensin metabolites were not reported by Camargo et al., and were thus inferred from the figures.

Supporting these findings, the two studies that measured systemic angiotensin responses in COVID-19 patients using ELISAs report markedly higher levels of both metabolites than two other studies that used LC-MS equilibrium analysis, another method that does not implement enzyme inhibitor cocktails (6, 7) (Figure 1A). Therefore, it's unlikely that the absence of enzyme inhibitor cocktails alone explains the high angiotensin levels described in both ELISA studies.

In an effort to reduce the difficulty of sample handling compared to gold-standard methods, LC-MS equilibrium angiotensin profiling analysis was recently developed. In this assay, enzyme inhibitor cocktails are not used, but the RAAS enzyme cascade is actually allowed to re-establish under controlled conditions by incubating samples for 1 h at 37°C. During this incubation, the formation and degradation of all angiotensin metabolites reaches an equilibrium, because angiotensinogen, the first (inactive) peptide in the RAAS cascade, is present in vast excess compared to its cleaved products, the active angiotensin metabolites. After reaching equilibrium, peptide levels are stabilized by adding enzyme inhibitors, after which LC-MS analysis is performed.

Importantly, because circulating RAAS enzymes remain active during incubation, the activity of membrane-bound RAAS enzymes is not assessed. This means LC-MS equilibrium analysis does not reflect true circulating angiotensin metabolite levels, but rather provides a detailed assessment of circulating RAAS-enzyme quantity and activity. Correspondingly, the two studies that implemented LC-MS equilibrium analysis report notably higher (equilibrium) angiotensin metabolite levels than the sole study that performed LC-MS with enzyme inhibitor cocktails, which likely accurately reflects true endogenous (circulating) content (Figures 1A,B) (6–8). Of note, while a discrepancy in angiotensin metabolite levels between the two studies using LC-MS equilibrium analysis also exists, this might well be explained by differences in disease severities, with reported SOFA scores of 6 (IQR 5–8) vs. 9 (IQR 8–11) at time of sampling (Figures 1A,B) (6, 7).

### Different conclusions of studies using liquid chromatography–mass spectrometry

As alluded to before, several research groups have assessed systemic angiotensin responses in COVID-19 patients using LC-MS approaches. The largest study to date (*n* = 126) employed an LC-MS equilibrium approach and found that equilibrium Ang-II levels increase with COVID-19 disease severity and are similar to those observed in critically ill patients with influenza (6). Another study using LC-MS equilibrium analysis reported lower levels of equilibrium Ang-II in severe COVID-19 patients than in healthy volunteers (7). In contrast, increased activity of the non-classical RAAS pathway, reflected by high circulating levels of equilibrium Ang-1–7 and soluble ACE2 as well as an increased Ang-1–7/Ang-II ratio, appears to be a finding unique to critically ill COVID-19 patients, reproduced in both studies that implemented an LC-MS equilibrium approach (6, 7). Recently, these results were confirmed in a study that used a classical LC-MS approach with enzyme inhibitors, providing even stronger evidence of enhanced non-classical RAAS activation in severe COVID-19 patients, also when membrane-bound RAAS enzymes are taken into account (8). Interestingly, increased Ang-1–7/Ang-II ratios are also present in the data of Camargo et al. (Figure 1B). In contrast to the absolute levels of angiotensin metabolites, these ratios are remarkably comparable to those reported by the LC-MS equilibrium studies (Figure 1B). Putatively, if Ang-II and Ang-1–7 are similarly affected by ongoing proteolysis after sampling, this could well explain why the Ang 1–7/Ang-II ratio appears to be less affected by differences in measurement methodology.

There remains extensive debate on how this non-classical RAAS activation might influence COVID-19 disease development. On one hand, membrane-bound ACE2 serves as the main point of viral entry, meaning high non-classical RAAS activity could reflect higher COVID-19 disease susceptibility (9). On the other hand, ACE2 and Ang-1–7 administration are known to protect from pulmonary injury in different murine ARDS models (10, 11). This could mean that non-classical RAAS activation actually reflects a protective mechanism, aimed at attenuating inflammation-induced pulmonary injury (10, 11).

Thus, while multiple studies report that Ang-II responses appear to increase according to COVID-19 disease severity, it is not the most pronounced RAAS-alteration in COVID-19 patients. Since all three studies using LC-MS approaches found changes in the markers corresponding with non-classical RAAS activation (i.e., Ang-1–7/Ang-II ratio) to be more pronounced depending on COVID-19 disease severity, we advocate their potential, in contrast to Ang-II alone, as a prognostic biomarker which should be investigated in prospective studies.

## Conclusion

Clearly, the increased availability of methods able to measure systemic angiotensin responses will lead to novel findings with potential clinical implications. However, there is marked variation in results of studies performed in the COVID-19-era, which can be attributed to differences in measurement methodology. This stresses the necessity of using assays that are both accurate and reproducible. Without such methods of quantification, new findings will carry the risk of causing confusion, rather than increasing our understanding on systemic angiotensin responses.

## Author contributions

DL: conceptualization (supporting) and writing—original draft (lead). MK: conceptualization (supporting) and writing—review and editing (supporting). PP: conceptualization (lead) and writing—review and editing (lead). All authors contributed to the article and approved the submitted version.

## Conflict of interest

The authors declare that the research was conducted in the absence of any commercial or financial relationships that could be construed as a potential conflict of interest.

## Publisher's note

All claims expressed in this article are solely those of the authors and do not necessarily represent those of their affiliated organizations, or those of the publisher, the editors and the reviewers. Any product that may be evaluated in this article, or claim that may be made by its manufacturer, is not guaranteed or endorsed by the publisher.
